# Modeling the economic impact of linezolid versus vancomycin in confirmed nosocomial pneumonia caused by methicillin-resistant *Staphylococcus aureus*

**DOI:** 10.1186/cc13996

**Published:** 2014-07-22

**Authors:** Dipen A Patel, Andrew F Shorr, Jean Chastre, Michael Niederman, Andrew Simor, Jennifer M Stephens, Claudie Charbonneau, Xin Gao, Dilip Nathwani

**Affiliations:** 1 Pharmerit International, 4350 East West Highway, Suite 430, Bethesda, MD 20814, USA; 2 Department of Pulmonary & Critical Care Medicine, Washington Hospital Center, 110 Irving St NW, Washington, DC 20010, USA; 3 Service de Réanimation Médicale Institut de Cardiologie Groupe, Hospitalier Pitié-Salpêtrière, 47-83 boulevard de l'Hôpital 75651, Paris Cedex 13, France; 4 Department of Medicine, Winthrop-University Hospital, 222 Station Plaza N, Suite 509, Mineola, NY 11501, USA; 5 Department of Microbiology, Sunnybrook Health Sciences Centre, B121-2075 Bayview Ave., Toronto, ON M4N 3M5, Canada; 6 Outcomes Research-Europe Infectious Diseases, Pfizer International Operations Specialty, Business Unit 23-25, avenue du Dr Lannelongue F-75668, Paris Cedex 14, France; 7 Ninewells Hospital & Medical School, Ward 42, East Block, Dundee DD19SY, UK

## Abstract

**Introduction:**

We compared the economic impacts of linezolid and vancomycin for the treatment of hospitalized patients with methicillin-resistant *Staphylococcus aureus* (MRSA)–confirmed nosocomial pneumonia.

**Methods:**

We used a 4-week decision tree model incorporating published data and expert opinion on clinical parameters, resource use and costs (in 2012 US dollars), such as efficacy, mortality, serious adverse events, treatment duration and length of hospital stay. The results presented are from a US payer perspective. The base case first-line treatment duration for patients with MRSA-confirmed nosocomial pneumonia was 10 days. Clinical treatment success (used for the cost-effectiveness ratio) and failure due to lack of efficacy, serious adverse events or mortality were possible clinical outcomes that could impact costs. Cost of treatment and incremental cost-effectiveness per successfully treated patient were calculated for linezolid versus vancomycin. Univariate (one-way) and probabilistic sensitivity analyses were conducted.

**Results:**

The model allowed us to calculate the total base case inpatient costs as $46,168 (linezolid) and $46,992 (vancomycin). The incremental cost-effectiveness ratio favored linezolid (versus vancomycin), with lower costs ($824 less) and greater efficacy (+2.7% absolute difference in the proportion of patients successfully treated for MRSA nosocomial pneumonia). Approximately 80% of the total treatment costs were attributed to hospital stay (primarily in the intensive care unit). The results of our probabilistic sensitivity analysis indicated that linezolid is the cost-effective alternative under varying willingness to pay thresholds.

**Conclusion:**

These model results show that linezolid has a favorable incremental cost-effectiveness ratio compared to vancomycin for MRSA-confirmed nosocomial pneumonia, largely attributable to the higher clinical trial response rate of patients treated with linezolid. The higher drug acquisition cost of linezolid was offset by lower treatment failure–related costs and fewer days of hospitalization.

## Introduction

Nosocomial pneumonia (NP) has been reported to be the second most frequent hospital-acquired infection in the United States [1]. Methicillin-resistant *Staphylococcus aureus* (MRSA) is responsible for a large number of cases of health-care–associated pneumonia, hospital-acquired pneumonia and ventilator-associated pneumonia [2,3]. A longitudinal study showed that the proportion of *Staphylococcus aureus* isolates that were methicillin-resistant (that is, MRSA) increased from 35.9% in 1992 to 64.4% in 2003 in ICUs in the United States [4]; however, more recent data from nine metropolitan areas suggest that the incidence rates have declined among patients with health-care–associated, community-onset or hospital-onset infections [5].

Despite the variation in incidence, MRSA infections remain a significant public health problem. MRSA-associated NP results in considerable patient morbidity, mortality and use of health-care resources with significant length of hospital stay [6-8]. The mean duration of hospitalization and associated costs of MRSA infections have been reported to be significantly higher than those of methicillin-susceptible *S. aureus* (MSSA) infections [9,10]. The majority of this cost difference can be attributed to excess hospitalization rather than to charges for antibiotic use, radiologic procedures or laboratory services.

Vancomycin and linezolid are the commonly recommended agents in clinical guidelines for the treatment of MRSA-related pneumonia [11,12]. In addition to these two agents, telavancin is the only other agent approved for the treatment of MRSA NP in the United States and Europe. Two large, prospective, randomized, double-blind trials demonstrated that linezolid (600 mg every 12 hours) was statistically noninferior to fixed-dose vancomycin (1 g twice daily) for the treatment of NP [13,14]. In a retrospective combined subgroup analysis of these two trials, researchers found significantly higher survival and clinical cure rates with linezolid treatment compared with vancomycin treatment [14]. Using *post hoc* data from the same studies, investigators have reported similar findings in patients with MRSA ventilator-associated pneumonia [15].

In a recent prospective, randomized, double-blind, controlled, multicenter study, specifically focused on MRSA-confirmed NP, researchers found greater clinical efficacy (defined as resolution of signs and symptoms, improved or lack of progression in chest imaging and no additional antibacterial treatment required) with linezolid than with adjusted-dose vancomycin [16]. That study’s sample size for the modified intent-to-treat population (MRSA-confirmed population) was 224 patients in each arm, with an end-of-study success rate of 57.6% for linezolid-treated patients and 46.6% for vancomycin-treated patients (95% confidence interval (CI) for differences from 0.5 to 21.6; *P* = 0.042). Linezolid was noninferior and statistically superior to vancomycin in end-of-treatment clinical and in end-of-treatment and end-of-study microbiologic outcomes. All-cause 60-day mortality rates were similar (15.7% for linezolid and 17.0% for vancomycin), as were the serious adverse event (SAE) rates.

Despite its higher acquisition costs, the overall cost for treating MRSA NP with linezolid may be lower because it is associated with better clinical outcomes compared with vancomycin. Yet, few researchers have investigated the costs associated with MRSA NP [17] and, in particular, the economic outcomes associated with treatments for MRSA NP. In two cost-effectiveness analyses based on a retrospective decision-analytic modeling approach, investigators found linezolid to be less costly and more efficacious than vancomycin for patients with suspected MRSA NP [18,19]. However, these earlier modeling studies either did not address the use of these agents in the US context [18] or were focused only on first-line therapy [19].

Our purpose in this economic analysis was to investigate the economic impact of improved clinical outcomes with linezolid compared with vancomycin in the treatment of hospitalized patients with MRSA-confirmed NP in the United States using a decision tree with a payer perspective and flexibility for real-world clinical conditions.

## Methods

### Model design

We conducted a cost-effectiveness analysis of intravenous (IV) linezolid compared with IV vancomycin for the treatment of MRSA NP in hospitalized adults, which was based on a decision tree modeling approach. The decision tree model was developed to capture first-line and second-line therapy. Because of the short-term window for the clinical management of an NP episode, the model time horizon was up to 4 weeks, which was validated by practicing physicians. This time horizon spans periods typical for ICU and general ward stays during first-line and second-line treatment [18,20,21]. A total payer perspective (assuming a *per diem* basis of payment) was considered in the base case analysis, which was comprehensive and comprised all inpatient and outpatient health-care costs (antibiotic and medical). Because this was an economic model in which we used only previously published data to create a hypothetical patient pathway, ethical approval and informed consent were neither applicable nor required.

The hypothetical model population was assumed to be similar to the population included in a recent phase IV, prospective, double-blind, controlled, multicenter, international clinical trial of IV linezolid (600 mg every 12 hours) or IV vancomycin (15 mg/kg every 12 hours, dose-adjusted based on trough levels and renal function) for the treatment of MRSA NP [16]. The full details of the characteristics and resource use for this MRSA-confirmed trial population have been reported previously [21,22] and include the following data: mean age 62 years, 69% white, 66% male, 75% mechanically ventilated, 87% had at least 1 day in the ICU and 63% were from the United States. The population used for analysis in the model was hospitalized adult patients with a confirmed MRSA NP diagnosis.

**Figure 1:**
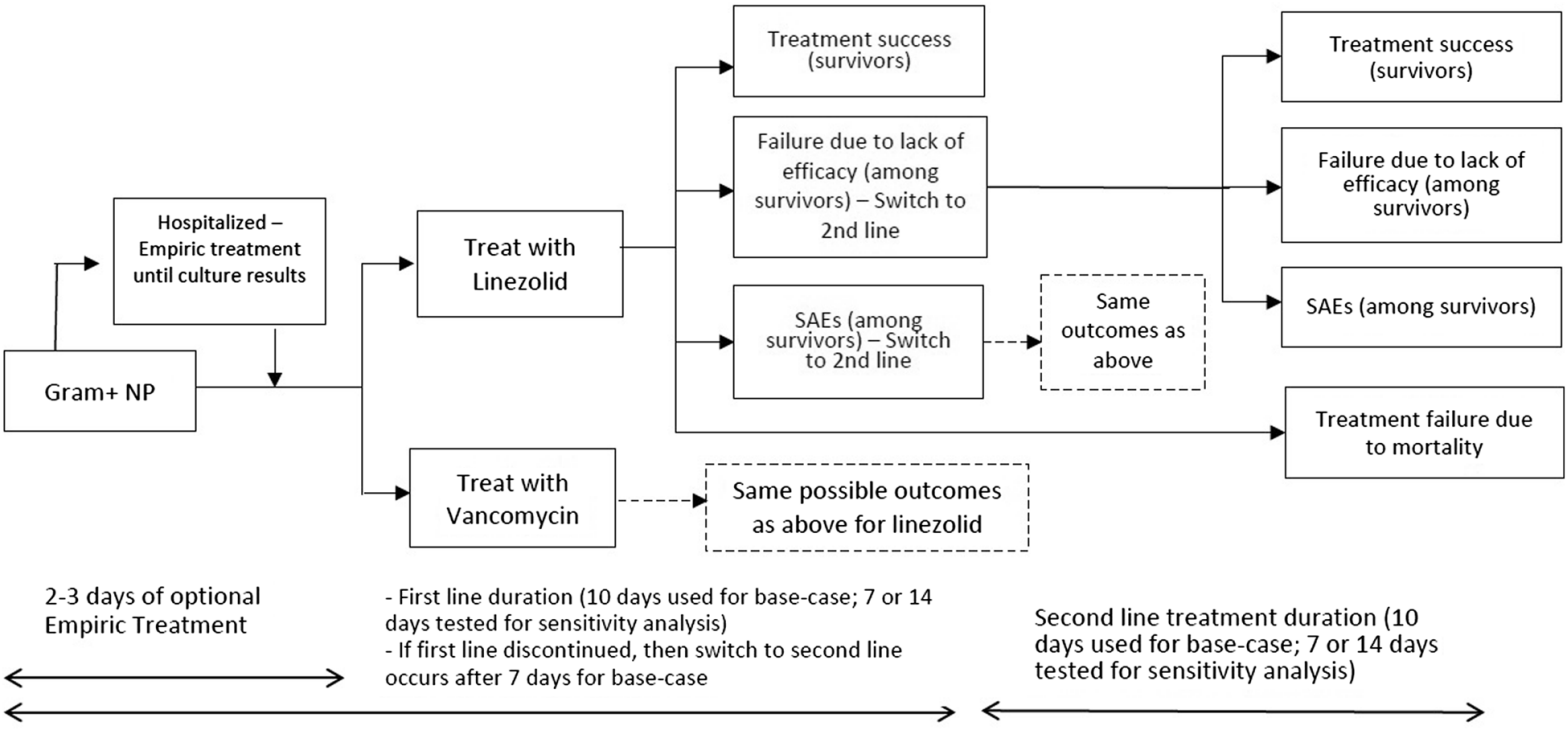
**Decision model tree.** NP, Nosocomial pneumonia; MRSA, Methicillin-resistant *Staphylococcus aureus*; SAEs, Serious adverse events.

Patients with suspected and/or confirmed Gram-positive NP could initially be treated with empiric IV antibiotic therapy (for example, vancomycin or linezolid in combination with ceftazidime, imipenem or piperacillin/tazobactam) for up to 3 days while laboratory confirmation of NP pathogen occurred (Figure 1). This empiric treatment pathway was not included in the base case analysis. Following confirmation of MRSA NP, the economic model analysis began and patients were placed on first-line treatment (vancomycin or linezolid) for 10 days (Figure 1). We focused on the component of treatment after MRSA confirmation when calculating cost-effectiveness, given the recent clinical trial data available [16] and because this is an important time point in clinical decision-making for reevaluation of the antibiotic treatment and coverage.Possible treatment outcomes associated with first-line therapy were (1) treatment success (defined as resolution of signs and symptoms of NP, improvement or lack of progression in chest imaging and no additional antibacterial treatment required among survivors), (2) failure due to lack of efficacy among survivors, (3) drug discontinuation due to SAEs and (4) failure due to death (Figure 1). A penalty, described in the section below, was assigned for patients whose treatment failed due to lack of efficacy or was discontinued due to SAEs.

Patients whose first-line treatment succeeded would finish their 10-day treatment duration and exit the model. In cases of any failure of first-line treatment, patients were switched to second-line treatment (for example, patients whose first-line treatment with linezolid failed were switched to second-line vancomycin, and vice versa) after 7 days, with the second-line treatment lasting 10 days. The model did not include a third-line treatment, given the lack of published data.

### Model inputs, outcomes and assumptions

In the base case scenario, the model was based primarily on recent MRSA NP clinical trial data [16] (Table 1). Linezolid and vancomycin were the main treatment comparators. In the base case analysis, we used 10-day treatment duration for the first- and second-line therapies. Data on length of hospital stay, inpatient and outpatient resource use and associated costs, and drug costs were obtained by analysis of the recent clinical trial and published literature (Tables 1 and 2) [16,21,23]. Key resources included in the model were days of antibiotic treatment, hospital general ward stay, hospital ICU stay, mechanical ventilator use, days on IV therapy, inpatient visits (to physician, attending and/or intensivist), inpatient laboratory work and physician office visits.

**Table 1:** **Model inputs on clinical and resource use data**^**a**^

	**Linezolid base case value (range**^**b**^**)**	**Vancomycin base case value (range**^**b**^**)**	**Distribution**^**b**^	**Source**
Efficacy and safety end points^c^,%				
Efficacy (in survivors)	54.8 (49.8^d^ to 66.7)	44.9 (35.5 to 52.9)	β	[16,20]
Mortality^e^	27.2	27.2	–	
SAEs leading to discontinuation^f^	1.8 (0^d^ to 5.2)	3.1 (0^d^ to 6.5)	β	[14,16]
Failure leading to discontinuation^g^	16.2	24.8	–	
Resource use				
Total days in hospital^h^	17.9 (13.9^d^ to 18.8)	18.6 (14.6^d^ to 20.1)	γ	[20,21]
Days of treatment^i^	10.0 (7 to 14)	10.0 (7 to 14)	Uniform	[16]
Days in ICU^h^	10.1 (6.1^d^ to 12.2)	10.6 (6.6^d^ to 16.2)	γ	[20,21]
Days on mechanical ventilation^h^	8.3 (4.3^d^ to 10.4)	8.1 (4.1^d^ to 14.3)	γ	[20,21]
Additional days in hospital due to SAE	1.7 (0 to 5)^d^	1.7 (0 to 5)^d^	γ	[21]
Additional days in hospital due to treatment failure	2 (0 to 5)^d^	2 (0 to 5)^d^	Uniform	Expert input
Number of days until switch to second-line after treatment failure/SAE with first- line	7 (5 to 10)^d^	7 (5 to 10)^d^	Uniform	Expert input
Days receiving IV antibiotic^h^	10.0	10.0	–	[16]
Antibiotic IV doses/day^h^	2.0	2.0	–	Product label
Physician/attending/intensivist visit (inpatient)/day^i^	1.0	1.0	–	Expert input
Lab work/wk^i,j^	7.0	8.0	–	Expert input

^a^IV, Intravenous; SAE, Serious adverse event. Clinical response rate for modified intent-to-treat population at end-of-study time point was used [16,21,23]. ^b^Ranges and distributions are provided for variables that were used in sensitivity analyses. ^c^Same clinical data were used for second-line treatment. ^d^This was an arbitrary assumption that was validated with expert opinion. ^e^Weighted average, because model assumes equal mortality due to lack of significant mortality difference (linezolid = 63 of 224; vancomycin = 59 of 224). ^f^Linezolid = 4 of 224; vancomycin = 7 of 224. ^g^Because this is a decision tree model, this probability was derived as [1 – (probability of efficacy + probability mortality + probability of SAEs leading to discontinuation)]. ^h^Data input for first line treatment only. ^i^Data input for first and second line treatment. ^j^Daily serum creatinine levels and complete blood count for both antibiotics and once-weekly serum vancomycin levels for vancomycin.

**Table 2:** **Model input data on unit costs of medical care (in 2012 US dollars)**^**a**^

**Cost inputs**	**Cost base case value (range**^**b**^**)**	**Source**
Inpatient cost per day (general ward)	$1,973.7 ($1,480.3 to $2,467.1)^c^	[24]
Inpatient cost per day (ICU)	$3,415.6 ($2,561.7 to $4,269.5)^c^	[24]
Mechanical ventilation per day	$225.2 ($168.9 to $281.5)^c^	[25]
Physician/attending/intensivist visit	$175.0	CPT 99233 [26]
Specialist inpatient visit	$251.2	CPT 99253 [26]
Laboratory test (serum creatinine levels)	$65.9	CPT 80069^d^[26]
Laboratory test (serum vancomycin levels)	$36.3	CPT 80202^e^[26]
Laboratory test (complete blood count)	$34.3	CPT 85025^d^[26]
Outpatient parenteral antibiotic therapy/day	$204.2	[27]
Injection costs for administration	$7.6	[28]
IV linezolid 600 mg	$114.6 ($86.0 to $143.3)^c^	[29]
IV vancomycin 1 g	$5.8 ($4.4 to $7.3)^c^	[29]

^a^CPT, Current procedural terminology; ID, Infectious disease; IV, Intravenous. All costs were adjusted to US dollars using medical care component of the US Consumer Price Index [24-29]. ^b^Ranges are provided for variables that were used in sensitivity analyses. γ-distribution was used for these variables for probabilistic sensitivity analysis. ^c^Arbitrary ±25% range was used. ^d^Based on testing once every day (while in hospital) for both linezolid and vancomycin. ^e^Based on testing once a week for only vancomycin.

In cases where a discontinuation due to an SAE or treatment failure occurred, patients were assumed to stay 1.7 or 2.0 additional days in the hospital, respectively, during first-line treatment compared with patients whose treatment was successful [18]. This additional length of stay was determined on the basis of *post hoc* analysis of recent clinical trial data [16,21,22] wherein bivariate analysis was conducted to compare length of stay in patients with or without moderate or severe adverse events and in patients with first-line treatment success versus failure. These values were further validated with expert opinion of the authors who reported the pertinent studies.

This study is primarily a cost-effectiveness analysis and not a cost–utility analysis, because the treatment effect of interest is drug efficacy (that is, proportion of patients successfully treated), instead of quality-adjusted life years (QALYs) or life-years (LYs). The latter two outcomes (QALYs and Lys) were not considered ideal for this analysis and hence are not reported, because the model uses a short-term duration and the trial data used for this model suggest equal mortality rates between linezolid and vancomycin [16]. As a result, there were negligible differences in QALYs and no differences in LYs between the treatment arms.

The key result outcomes of this analysis, which are reported in the Results section, are total costs and effectiveness proportion for the two treatments, total cost per successfully treated patient for each treatment (calculated as ratio of total costs and total effectiveness) and incremental cost-effectiveness ratio (ICER), calculated as the difference in costs between treatments divided by the difference in the proportion of successfully treated patients receiving linezolid versus vancomycin.

The following key assumptions were made in the model:

● Every patient received treatment as long as they were hospitalized, and all patients were on IV therapy during their hospital stay.

● In the absence of published data for second-line treatment, the clinical inputs for second-line treatment were the same as those used for first-line treatment [18].

● Because we used the 60-day mortality rates reported in the clinical trial [16], which represented total mortality and included deaths associated with first-line and second-line treatment, mortality occurred only at the end of first-line treatment to avoid overestimation attributable to double-counting. Because the first-line mortality rates did not statistically differ between linezolid and vancomycin in the clinical trial, these rates were considered the same in the model.

● There were no patient dropouts due to failure or SAEs after first-line treatment.

● Patients whose second-line treatment failed and those who had SAEs were deemed to have completed the duration of therapy because no third-line therapy was available.

● Although the mean ICU stay was 10 days, ICU stay was considered to be 7 days if treatment duration was 7 days and the patient’s first-line treatment succeeded. Alternatively, if the treatment duration was 14 days, then the ICU stay would be 10 days and the remaining 4 days would be in the general ward.

### Sensitivity analyses

Univariate (one-way) sensitivity analysis was conducted to assess the impact of model uncertainties and the robustness of our analysis. Key model parameters were varied individually within the predefined sensitivity ranges (Tables 2 and 3), and ICERs were recorded. A published source was used for ranges whenever possible. In the absence of strong published data, an arbitrary range was used (such as ±4 days for length of stay or ±25% for costs). The results are presented in the form of a tornado diagram, with the variables stacked in decreasing order of impact on the ICER.

**Table 3:** **Detailed cost results of the base case scenario**^**a**^

**Cost items per patient**	**Linezolid**	**Vancomycin**
Drug treatment	$2,189	$746
Drug administration	$172	$182
*Inpatient drug cost*	*$2,361*	*$928*
ICU	$34,217	$34,728
General ward	$2,451	$3,524
Mechanical ventilation	$1,869	$1,824
Physician/attending visit	$1,970	$2,091
Lab work	$1,137	$1,245
SAE/failure costs	$2,162	$2,651
*Inpatient medical cost*	*$43,807*	*$46,064*
**Total costs**	**$46,168**	**$46,992**

^a^SAE, Serious adverse event.

A probabilistic sensitivity analysis (PSA) was also performed, wherein all parameters were varied simultaneously within their range using 10,000 second-order Monte Carlo simulations. γ-distribution was specified for resource use and cost variables, and β-distribution for probability variables.

## Results

### Base case analysis

Under the model base case settings (with no empiric treatment, a 10-day treatment duration, and discontinuation or switch of therapy possible after 7 days), the total inpatient (medical plus drug) costs were $46,168 for linezolid and $46,992 for vancomycin (Table 3). Although the drug costs were $1,433 higher with linezolid compared with vancomycin, the medical costs associated with linezolid were $2,256 lower with linezolid than with vancomycin. Overall, treatment with linezolid was associated with lower total costs (by a mean of $824) and greater effectiveness (+2.7% absolute difference in proportion of successfully treated patients) compared with vancomycin. The expected proportions of successfully treated patients were 62.9% and 60.2% for linezolid and vancomycin, respectively. Factoring in these expected success rates, the total costs per successfully treated patient were predicted to be $73,420 (linezolid) and $78,073 (vancomycin), for a total cost savings of $4,653. Thus, the ICER (in this case, incremental cost per successfully treated patient) was in favor of linezolid compared with vancomycin (that is, linezolid dominated vancomycin), owing to linezolid’s lower total costs and greater efficacy in successfully treating patients.

We calculated that, within the model, approximately 80% of the total treatment costs were attributable to hospital stay, primarily ICU costs, because each patient stayed at least 10 days (plus additional days if first-line therapy failed) in the ICU and the cost per day of ICU stay in the United States is very high. General ward costs were higher with vancomycin compared with linezolid, because, even though the length of stay in the hospital was comparable between treatments, there was a higher percentage of patients for whom vancomycin failed as first-line therapy and thus were transitioned to second-line treatment and had an associated longer general ward stay. Moreover, the higher percentage of vancomycin-treated patients requiring second-line therapy may have led to marginally higher costs for additional physician visits and laboratory work. However, drug therapy, physician visits, laboratory tests and SAEs and/or treatment failure each accounted for no more than 5% of the total costs (Table 3).

### Sensitivity analysis

**Figure 2:**
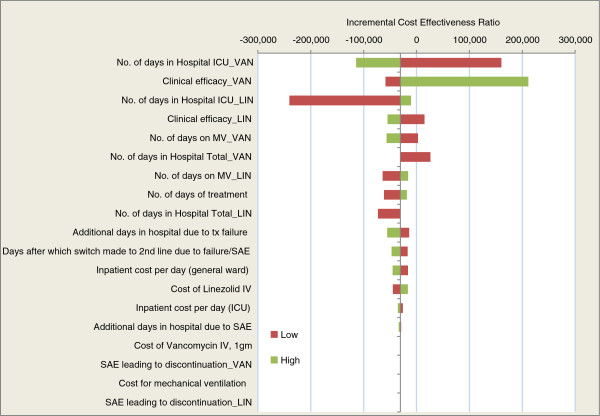
**One-way sensitivity analyses of key parameters (Tornado diagram).** IV, Intravenous; LIN, Linezolid; MV, Mechanical ventilation; SAE, Serious adverse events; tx, Treatment; VAN, Vancomycin.

**Figure 3:**
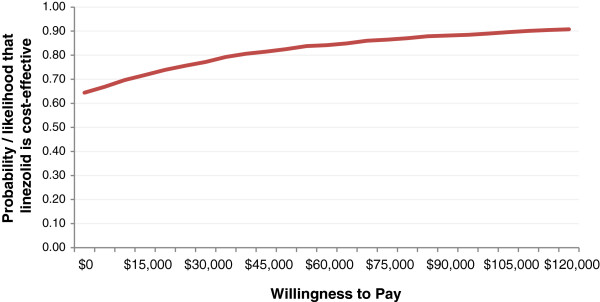
Cost-effectiveness acceptability curve of linezolid versus vancomycin.

The results of one-way sensitivity analysis (as seen in the Tornado diagram in Figure 2) demonstrated variables that had the greatest impact on the model results. The ICERs ranged from a low of about − $240,000, when ICU stay with linezolid was at its lower value of 6.1 (suggesting a dominant scenario for linezolid), to a high of around $210,000, when the clinical efficacy of vancomycin was at its higher value of 52.9% and $160,000, when ICU stay with vancomycin was at a low of 6.6 days. (These ICERs can be considered greater than the acceptable willingness-to-pay (WTP) threshold, making vancomycin the cost-effective option.) There is no clearly defined WTP threshold for successful treatment of one patient, and hence different WTP values were tested in the PSA cost-effectiveness acceptability curve.A cost-effectiveness acceptability curve generated from a PSA is presented in Figure 3. This plot displays the percentages for linezolid being more cost-effective compared to vancomycin at the different WTP thresholds. Linezolid had a 64.4% likelihood of being cost-effective at a WTP threshold of $0 and a 90.8% chance at a WTP threshold of $120,000.

## Discussion

This economic decision tree analysis is the first, to our knowledge, to mirror real-world clinical conditions by allowing for a switch of therapy if needed (that is, it models first- and second-line treatment) and allowed us to assess the impact of varying treatment parameters, including treatment duration. To our knowledge, no other published studies of NP have researchers accounted for these factors from a US health-care payer perspective. Our results with this model show that linezolid is a cost-effective alternative to vancomycin for the treatment of MRSA-confirmed NP, owing primarily to the higher clinical response rate of linezolid-treated patients compared with vancomycin-treated patients. The higher acquisition cost of linezolid was offset by lower costs of treatment failure and SAEs, as well as fewer days spent in the hospital, when we accounted for combined first-line and second-line therapies. Only direct medical costs were included in the model, with a distinction made between inpatient and outpatient costs. For NP, inpatient costs accounted for the largest proportion of overall costs.

Linezolid was a more cost-effective treatment option in the majority of one-way sensitivity analyses (vancomycin was cost-effective only under two scenarios: low ICU stay and high vancomycin efficacy rate) and under varying WTP thresholds in PSA. Length of ICU stay and clinical efficacy rate appeared to be the most sensitive variables in one-way analysis, with the greatest impact on the ICER. This was expected, especially with regard to the length of ICU stay, because ICU stay *per diem* is very expensive in the United States and the cost of ICU stay accounts for the largest proportion of total treatment cost.

Our results are consistent with those reported in two previous cost-effectiveness analyses in which investigators found therapy initiated with linezolid to be less costly and more efficacious than vancomycin for patients with suspected MRSA NP [18,19]. Mullins *et al*. applied a retrospective decision-analytic model to pooled efficacy data derived from two clinical trials and health plan hospital claims and determined hospital costs for US patients with suspected NP. When median daily hospital charges and mean treatment durations were factored in, total hospitalization charges were estimated to be $32,636 for linezolid treatment compared with $32,024 for vancomycin treatment. The ICER for linezolid per life saved was $3,600. However, they based their efficacy estimates on a small sample of patients with MRSA NP (*N* = 160) and examined the cost-effectiveness of only first-line linezolid or vancomycin treatment.

In a German cost-effectiveness analysis [18], the researchers used a decision-analytic model based on previously published clinical data [14] and found higher clinical cure and survival rates with linezolid, but at a small incremental cost compared with vancomycin, resulting in acceptable ICERs of cost per death avoided and cost per patient cured [18]. From a clinical standpoint, they demonstrated that linezolid had better efficacy than vancomycin for the treatment of MRSA NP (on the basis of trial data specifically in MRSA-confirmed patients), with fewer patients requiring a switch to second-line therapy. The longer hospital stays associated with switching from vancomycin as first-line treatment to a second-line therapy required additional resource use, including physicians’ and other health-care professionals’ time that could have been spent treating other patients.

Our present economic analysis included patients who received optional empiric therapy (2 days) followed by first- and/or second-line treatment once MRSA was confirmed, with the empiric treatment costs not included in the presented scenarios. Costs, therefore, were not considered in patients who did not have MRSA infection. In clinical practice, initiation with empiric antibiotic treatment is started as soon as MRSA is suspected, and antibiotic treatment success and the related costs of empiric therapy are determined by how well MRSA is predicted and by the proportion of patients with MRSA in the treated population. Our present analysis therefore does not include the costs of initial empiric therapy and the harm that comes from (1) not covering MSSA by using only MRSA coverage, (2) choosing vancomycin and the possibility of renal toxicity developing in a patient without MRSA and (3) not starting empiric therapy with either drug and having a delay in starting appropriate therapy until after culture results have been confirmed. Although we did not address these clinical aspects in our model, they are relevant and important and should be explored in future studies.

Vancomycin and linezolid are the most commonly recommended and prescribed treatment options for MRSA NP [11,12]. Vancomycin has been the mainstay generic for decades; however, challenges with tissue penetration at the site of infection, therapeutic drug monitoring and increased risk for renal dysfunction in NP patients makes the use of this agent more difficult in critically ill patients. In the economic analysis presented here, we used the recent and only clinical trial data specifically designed to evaluate clinical success in the treatment of patients with MRSA NP [16]. To date, linezolid is the only agent to have proven better clinical success rates in NP than vancomycin in a MRSA-only population. Linezolid is sold as ZYVOX and is under patent in the United States until the end of 2014; thus, use of this agent may increase further with the introduction of generic versions. However, there is another oxazolidinone drug for nosocomial pneumonia currently under development, tedizolid, in ongoing phase III trials. A newer glycopeptide, telavancin, became available in late 2013 for gram positive NP, and while the phase III trials included MRSA patients, the studies were not specifically designed to examine clinical success in the MRSA-only population. Thus, the only MRSA-specific NP study to date is the Wunderink *et al*. study [16], on which our economic analysis is based.

Our study has limitations. In the model’s base case scenario, we considered the conditions under which the Wunderink clinical trial was performed [16], which may differ in real-life US clinical practice. Further, because the Wunderink trial enrolled US patients, the results may not be applicable to scenarios outside the United States. The model included only first-line and second-line treatments, not potential later treatment options. However, this is consistent with other published models [18] and is justifiable because the majority of the resources used and outcomes witnessed were within the first two lines of therapy. In the model, we estimated direct costs only and did not include indirect costs related to lost productivity incurred as a result of the length of hospital stay, convalescence or early mortality.

We used 60-day mortality data, calculated as a weighted average of the 60-day mortality rates for the modified intent-to-treat population in the clinical trial [16], which were the best available “proxy” data for this 4-week model, given that the difference between 30 and 60 days was found to be small based on the survival curve derived from the study. In addition, mortality rates were not statistically different in the clinical trial; thus, a cost per LY saved calculation was less relevant, given that the trial was never designed to show a difference in mortality. In fact, patients could have received up to 2 days of vancomycin before being randomized to the study drugs; thus, patients doing poorly on vancomycin would have been less likely to be enrolled in the study, where the chance of being randomized to vancomycin was 50–50. Instead of focusing on LYs, we used “proportion of successfully treated patients” instead of QALYs as the efficacy measure in this model, which could be considered a drawback, especially because there is no clearly defined ICER threshold per successfully treated patient. However, we think that successful treatment is a clinically important efficacy measure for NP, and hence it can be argued to be relevant for this model.

## Conclusion

Our US health-care system economic model using recent MRSA-specific clinical trial data shows that treatment with linezolid is less costly and more efficacious than treatment with vancomycin for MRSA-confirmed NP. Cost savings with linezolid were derived largely from lower treatment failure rates, fewer days of hospitalization and lower incidence of renal failure. We found our findings to be consistent in sensitivity analyses. In future analyses, researchers should use other country costs and resource-use data to test result generalizability and could model the empiric treatment phase before MRSA confirmation.

## Key messages

● Linezolid is likely to be a cost-effective alternative compared to vancomycin for the treatment of MRSA NP, primarily owing to the former’s better clinical success rate.

● Higher drug costs for linezolid are offset by lower overall medical costs due to fewer treatment failures and fewer serious adverse events, such as renal failure, as well as fewer days spent in the hospital, when accounting for combined first-line and second-line therapies.

● For MRSA NP, inpatient costs accounted for the largest proportion of overall costs.

## Abbreviations

AE: Adverse event; CPT: Current procedural terminology; ICER: Incremental cost-effectiveness ratio; ID: Infectious disease; IV: Intravenous; MRSA: Methicillin-resistant *Staphylococcus aureus*; NP: Nosocomial pneumonia; PSA: Probabilistic sensitivity analysis; SAE: Serious adverse event.

## Competing interests

This study was sponsored by Pfizer. The following authors declare receiving lecture or advisory board or research grant support: ASi, AFS, DN, MN and JC (Pfizer); AFS, DN and JC (Astellas Pharma); DN (AstraZeneca); AFS, DN and MN (Bayer); JC (Nektar-Bayer); DN (Basilea Pharmaceutica); JC (B•R•A•H•M•S); AFS, DN and MN (Cubist Pharmaceuticals); DN (Durata Therapeutics); AFS (Forest Laboratories); JC (Janssen-Cilag Pharma); MN (Merck); JC and MN (Sanofi-Aventis); ASi (Sunovion Pharmaceuticals Canada); AFS (Tetraphase Pharmaceuticals); AFS (Theravance Biopharma); and AFS and JC (Trius Therapeutics). CC is an employee of Pfizer. DAP, JMS and XG are employees of Pharmerit. Pharmerit received research funding from Pfizer to develop the model. No funding was provided to the authors for manuscript development. Editorial and medical writing support was provided by Ray Beck, Jr, PhD, of Engage Scientific Solutions and was funded by Pfizer. The authors declare that they have no competing interests other than those described here.

## Authors’ contributions

DP was responsible for model conceptualization and design, development of the model inputs and assumptions, programming, analyses, review and interpretation of results and manuscript writing. AFS, ASi, MN and DN were responsible for model conceptualization, design and assumptions; review and interpretation of results; and critical revision of the manuscript. JS was responsible for model conceptualization and design, development of the model inputs and assumptions, programming, analyses, review and interpretation of results and manuscript writing. CC was responsible for model conceptualization and design, financial support, review and interpretation of results and development of the manuscript. XG was responsible for model conceptualization and design, development of the model inputs and assumptions, programming, analyses, review and interpretation of results and critical revision of the manuscript. All authors read and approved the final manuscript.
